# A knockout cell library of GPI biosynthetic genes for functional studies of GPI-anchored proteins

**DOI:** 10.1038/s42003-021-02337-1

**Published:** 2021-06-23

**Authors:** Si-Si Liu, Yi-Shi Liu, Xin-Yu Guo, Yoshiko Murakami, Ganglong Yang, Xiao-Dong Gao, Taroh Kinoshita, Morihisa Fujita

**Affiliations:** 1 Key Laboratory of Carbohydrate Chemistry and Biotechnology, Ministry of Education, School of Biotechnology, Jiangnan University, Wuxi, Jiangsu China; 2 Research Institute for Microbial Diseases, Osaka University, Suita, Osaka Japan; 3 WPI Immunology Frontier Research Center, Osaka University, Suita, Osaka Japan

**Keywords:** Glycobiology, Glycobiology

## Abstract

Over 100 kinds of proteins are expressed as glycosylphosphatidylinositol (GPI)-anchored proteins (GPI-APs) on the cell surface in mammalian cells. GPI-APs possess unique properties in terms of their intracellular trafficking and association with lipid rafts. Although it is clear that GPI-APs play critical roles in various biological phenomena, it is poorly understood how the GPI moiety contributes to these mechanisms. More than 30 genes are involved in the correct biosynthesis of GPI-APs. We here constructed a cell library in which 32 genes involved in GPI biosynthesis were knocked out in human embryonic kidney 293 cells. Using the cell library, the surface expression and sensitivity to phosphatidylinositol-specific phospholipase C of GPI-APs were analyzed. Furthermore, we identified structural motifs of GPIs that are recognized by a GPI-binding toxin, aerolysin. The cell-based GPI-knockout library could be applied not only to basic researches, but also to applications and methodologies related to GPI-APs.

## Abstract (web summary)

Liu et al. construct a knock out cell library of genes involved in the biosynthesis of glycosylphosphatidylinositol (GPI)-anchored protein (GPI-APs) from HEK-293 cells and analyzed the surface expression and sensitivity to phosphatidylinositol-specific phospholipase C. Furthermore, the authors also identify structural motifs of GPIs that are recognized by a GPI-binding toxin, aerolysin.

## Introduction

Various types of proteins, including single-membrane-spanning or multi membrane-spanning transmembrane proteins, lipidated proteins, and peripheral proteins, are localized on the cell surface. A number of proteins are covalently bound with a glycolipid, named glycosylphosphatidylinositol (GPI), and are localized at the plasma membrane. GPI anchoring of proteins is one of the common posttranslational modifications in eukaryotes^1–4^. GPI confers unique properties to modified proteins. GPI-anchored proteins (GPI-APs) are the major proteins that associate with lipid rafts, which are dynamic membrane microdomains composed of sphingolipids and cholesterol^5^. The association with lipid rafts regulates GPI-APs in polarized transport, endocytosis, and signal transduction in mammalian cells^6–9^.

The core structure of GPI is conserved and consists of EtNP-6-Man-α1,2-Man-α1,6-Man-α1,4-GlcN-α1,6-inositol-phospholipid (EtNP, ethanolamine-phosphate; Man, mannose; GlcN, glucosamine) among eukaryotic species, whereas the lipid moieties and glycan side chains are different^10^. In mammalian cells, several side chain modifications on GPI-glycan structures are found. The first Man (Man1) is modified with a side chain, EtNP. The fourth Man (Man4) can be attached to the third Man (Man3) via an α1,2-linkage^11^. In some proportions of mammalian GPI-APs, *N*-acetylgalactosamine (GalNAc) is modified to the Man1 through the β1,4-linkage^12,13^, and it can be further modified by β1,3-galactose (Gal)^14^ or β1,3-Gal with α2,3-sialic acid^15,16^. The GPI lipid portion is 1-alkyl-2-acyl-glycerol or diacylglycerol in mammals.

The biosynthesis of GPI is essential for embryonic development, neurogenesis, immune responses, and fertilization. Pathological congenital mutations in GPI biosynthetic genes cause inherited GPI deficiencies (IGDs), which are characterized by intellectual disability, epileptic seizures, hypotonia, and facial dysmorphisms^17^. Acquired mutations in a GPI biosynthesis gene in hematopoietic stem cells lead to paroxysmal nocturnal hemoglobinuria, in which hemolysis of red blood cells occurs by self-activation of the complement system^18^. On the other hand, the expression of GPI-APs, such as carcinoembryonic antigen (CEA)^19^, mesothelin^20^, folate receptor^21^, glypican-3^22^, and CD52^23^ is upregulated in various cancer cells, and such GPI-APs are utilized as biomarkers and therapeutic targets.

GPI biosynthesis and transfer to proteins are carried out in the endoplasmic reticulum (ER). After GPI is transferred to proteins, the lipid and glycan parts of GPI moieties are processed at the ER and the Golgi during GPI-AP transport. Some GPI-APs are cleaved at the GPI moiety by GPI-cleaving enzymes (GPIases)^24^. There are at least 23 steps in which 33 genes (including 21 phosphatidylinositol-glycan (PIG) genes and 6 post-GPI attachment to proteins (PGAP) genes) are involved for correct GPI-AP biogenesis. Among them, it was reported that defects in 23 GPI biosynthetic genes cause IGDs^25^. Although the genes responsible for reactions in most steps were identified, it is still not clear how GPI biosynthesis is regulated and which GPI structures are required for these functions.

Here, we performed a systematic genetic disruption of GPI biosynthetic genes in a human embryonic kidney 293 (HEK293) cell line, providing a knockout gene cell library with different GPI-anchor biosynthesis capabilities (GPI-KO cell library). We used the CRISPR-Cas9 system to construct the library and systematically analyzed the expression and sensitivity to phosphatidylinositol-specific phospholipase C (PI-PLC) of GPI-APs. We proved that the GPI-KO cell library can be applied to determine toxin recognition sites and the unique biological characteristics of prion proteins. Our GPI-KO cell library is a sustainable resource for exploring various applications and methodologies of GPI-AP biology.

## Results

### GPI-AP synthesis capacity of HEK293 cells

HEK293 cells, which are widely used in both basic and applied studies, were used to construct a GPI-KO cell library. We obtained the expression of genes involved in the biosynthesis, processing, and transport of GPI-APs in HEK293 cells using RNA-seq (Supplementary Fig. 1a). All the genes required for GPI-AP biogenesis were expressed in HEK293 cells, whereas the expression of some genes, such as *PIGY*, *PIGZ*, and *B3GALT4*, was limited. In addition, the expression profiles of genes encoding GPI-APs were analyzed. At least 67 GPI-APs were expressed (TPM value ≥ 1) in HEK293 cells (Supplementary Fig. 1b). We detected endogenous GPI-APs expressed on the surface of HEK293 cells by flow cytometry (Supplementary Fig. 2a). Several GPI-APs that were not expressed in HEK293 cells were exogenously expressed, and their surface expression was detected (Supplementary Fig. 2b), suggesting that HEK293 cells could be used for the study of the biological characteristics of GPI-APs.

### Genetic disruption of GPI biosynthesis in HEK293 cells

**Fig. 1:**
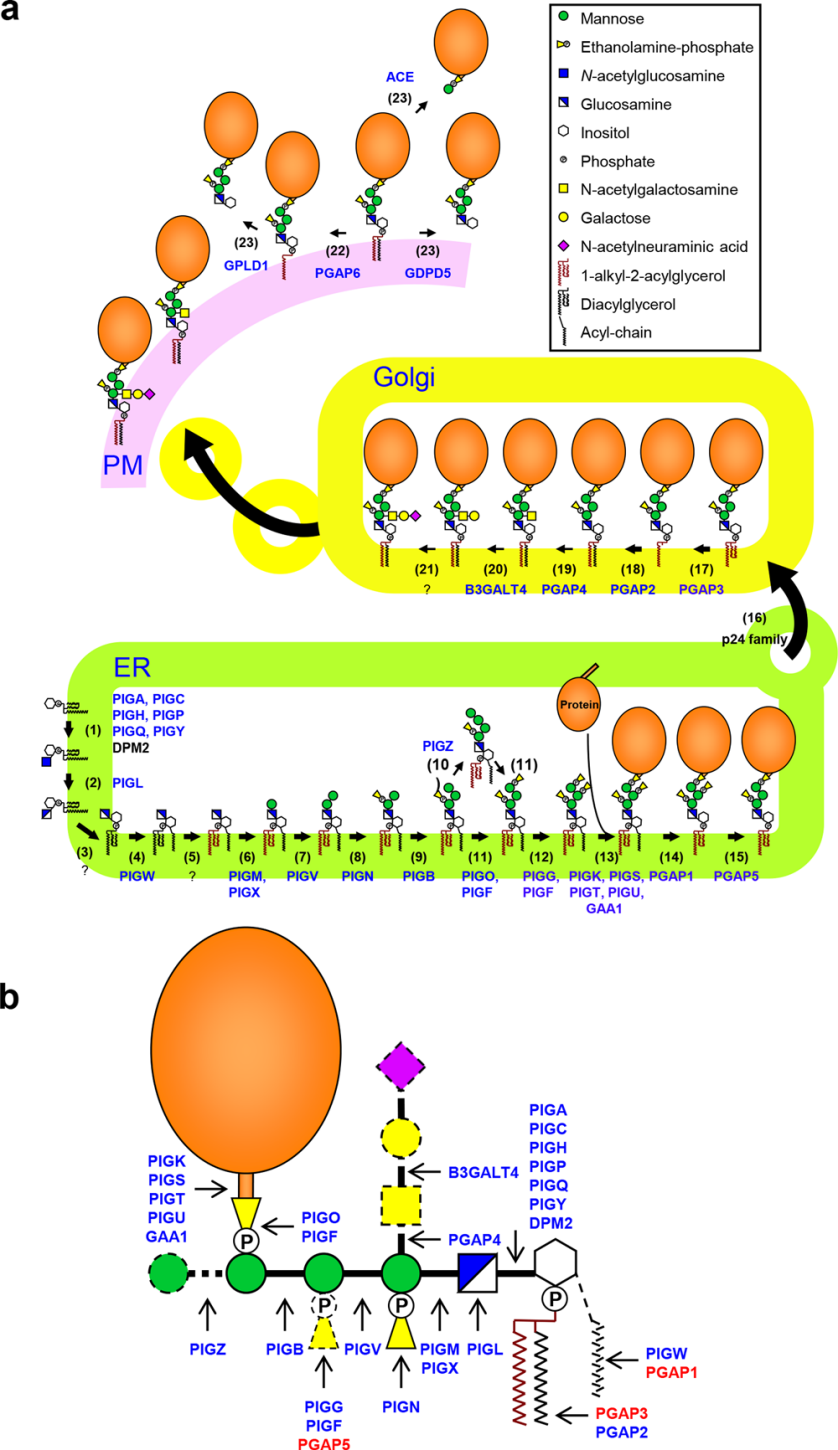
The biosynthesis of GPI-APs in mammalian cells. **a** GPI biosynthesis is carried out in the ER, Golgi, and the cell surface through a series of catalytic reactions. It starts with the transfer of GlcNAc to PI. The first two steps occur on the cytoplasmic side of the ER, and then the modification of polysaccharides and lipids occurs on the luminal side of the ER and Golgi. Proportions of GPI-APs further undergo side chain modification. Gene products that were knocked out in this study are highlighted in blue. **b** Schematic representation of a human GPI-AP structure. Gene products required for the synthesis of the GPI structure are shown in blue, and gene products that remove the structure are shown in red. The solid lines of the GPI structure indicate the core structure in human cells, whereas the dotted lines indicate accessory structures observed in human GPI-APs.

Using similar strategies to those employed for the knockout cell libraries of N-glycosylation^26^ and glycosaminoglycans (GAGs)^27^, genes encoding GPI biosynthesis were knocked out using the CRISPR–Cas9 system (Supplementary Fig. 3). We systematically designed knockout constructs targeting genes involved in GPI biosynthesis and validated the guide RNAs (gRNAs) for disruption (Fig. 1 and Supplementary Table 2). Two targets were selected on one exon of each gene and designed to allow knockout confirmation by eliminating the sequence on that exon. Then, using the validated gRNAs, we constructed 32 gene knockout cells (Fig. 1a), generating a GPI-KO cell library.

#### Initial steps of GPI biosynthesis

**Fig. 2:**
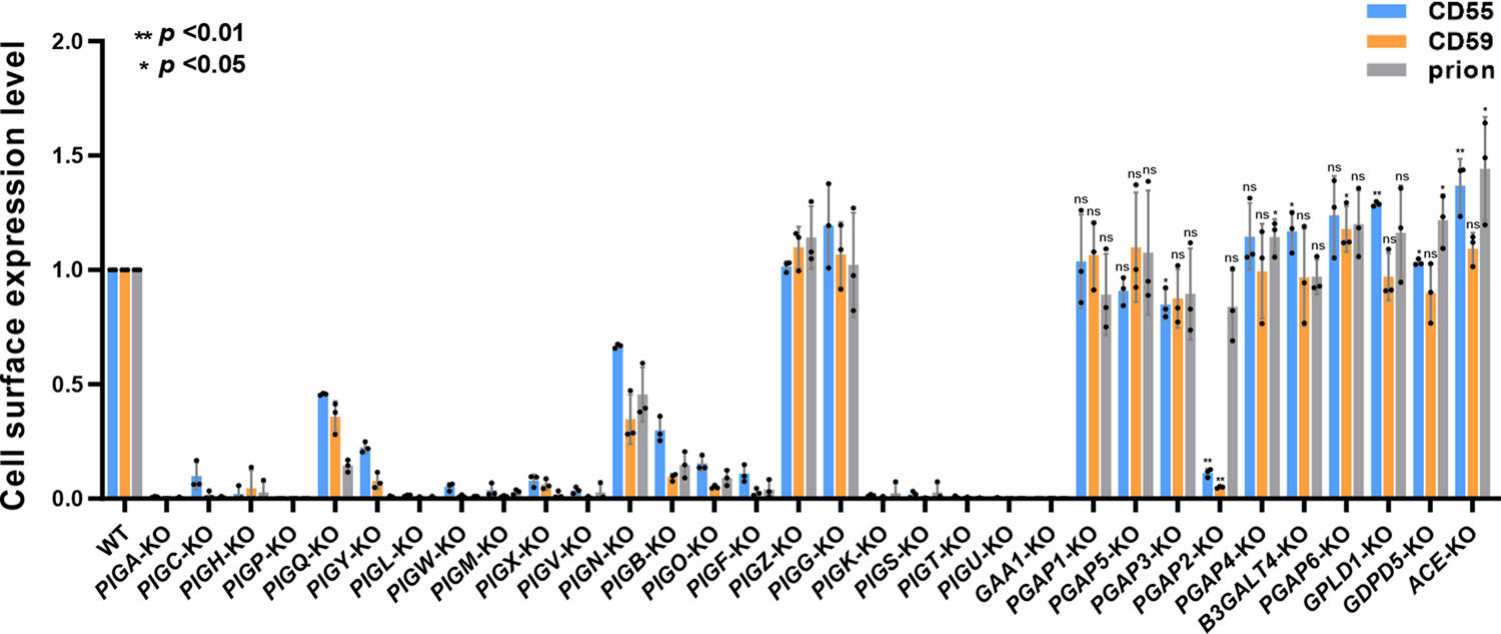
GPI-AP expression on the cell surface in the GPI-knockout cell library. Cell surface expression of three endogenous GPI-APs, CD55, CD59, and prion, was detected by flow cytometry. The expression level of GPI-APs in WT cells was set as 1, and the relative mean ± SD values from three independent experiments were displayed in a bar plot. CD55, blue; CD59, orange; prion, gray. *p < 0.05; **p < 0.01 according to an unpaired Student’s *t* test. ns not significant.

GPI biosynthesis starts with the addition of N-acetylglucosamine (GlcNAc) to PI at the cytosolic side of the ER membrane. GPI-GlcNAc transferase (GPI-GnT), composed of six core subunits, PIGA, PIGC, PIGH, PIGP, PIGQ, and PIGY, mediates GlcNAc to PI^28^ (Fig. 1a, Step 1). KO of *PIGA*, *PIGC*, *PIGH*, and *PIGP* completely lost GPI-AP expression, whereas KO of *PIGQ* and *PIGY* maintained weak expression of GPI-APs (Fig. 2 and Supplementary Fig. 4a), suggesting that KO of regulatory subunits retained GPI-GnT activity.

PIGL is required for the deacetylation of GlcNAc-PI to generate GlcN-PI^29^ (Fig. 1a, Step 2). GlcN-PI is then flipped into the luminal side of the ER. Inositol acyltransferase, PIGW, catalyzes the addition of an acyl chain to the 2-position of the inositol ring on GlcN-PI to form GlcN-(acyl)PI^30^ (Fig. 1a, Step 4). KO of these genes completely eliminated the synthesis of GPI-APs (Fig. 2 and Supplementary Fig. 4a).

#### GPI-mannosyltransferases (GPI-ManTs) and GPI-EtNP transferases

The complex composed of PIGM^31^ and PIGX^32^ (GPI-ManT-I) (Fig. 1a, Step 6), PIGV^33^ (GPI-ManT-II) (Fig. 1a, Step 7), PIGB^34^ (GPI-ManT-III) (Fig. 1a, Step 9), and PIGZ^11^ (GPI-ManT-IV) (Fig. 1a, Step 10) catalyzes the transfer of the first, second, third, and fourth Man to the GPI intermediate. PIGM and PIGX make a complex for GPI-ManT-I. The surface expression of GPI-APs was completely removed by KO of *PIGM*, which encodes the catalytic subunit of GPI-ManT-I. On the other hand, weak expression of GPI-APs remained after KO of *PIGX*, which encodes the regulatory subunit. KO of *PIGV* also completely removed the surface expression of GPI-APs. In contrast, KO of *PIGB* left some GPI-AP expression. The fourth Man modification by PIGZ was nonessential and its KO did not affect the biosynthesis of GPI-APs (Fig. 2). Since both PIGB and PIGZ are α1,2-ManTs, we knocked out *PIGZ* in *PIGB*-KO cells to check the redundancy. Even if *PIGZ* was knocked out in *PIGB*-KO cells, the expression of CD55 was not changed (Supplementary Fig. 4b), suggesting that PIGB and PIGZ do not have redundancy. Since it was shown that other ER-localized ManTs such as ALG3, 9, and 12, which are required for the biosynthesis of lipid-linked oligosaccharides, have strict specificity^35^, it would not be possible that those ManTs have redundancy to GPI-MTs. Instead, a GPI-anchor structure, in which a protein is attached to the second EtNP on Man2, was found^36^. Therefore, it is possible that proteins linked to the second EtNP of GPI are expressed in *PIGB*-KO cells.

Three EtNPs are added to Man1, Man3, and Man2 mediated by PIGN^37^ (GPI-EtNP transferase-I) (Fig. 1a, step 8), PIGO and PIGF complex^38,39^ (GPI-EtNP transferase-III) (Fig. 1a, step 11), and PIGG and PIGF complex^40^ (GPI-EtNP transferase-II) (Fig. 1a, step 12), generating a complete GPI precursor consisting of EtNP-Man-(EtNP)Man-(EtNP)Man-GlcN-(acyl)PI. GPI-APs were still expressed in *PIGN*-deficient cells, although the expression level was reduced to approximately 50%. In *PIGG*-KO cells, the expression of GPI-APs was normal, whereas it was extremely low in *PIGO*-deficient cells (Fig. 2). Although PIGB and PIGO mediate the addition of Man3 and EtNP to Man3 for protein binding, respectively, the KO cells did not completely lose GPI-AP expression. As mentioned above, we found a GPI-anchor structure in which a protein is attached to the second EtNP on Man2^36^. In *PIGO*-KO cells, a proportion of proteins utilize the second EtNP for GPI anchoring.

#### GPI attachment to proteins

GPI-transamidase (GPI-TA) is a multisubunit complex containing five subunits: PIGK^41^, GAA1^42^, PIGT^43^, PIGS^43^, and PIGU^44^. It recognizes the C-terminal GPI attachment signal of proteins^45^. PIGK is a catalytic subunit that cleaves the GPI attachment signal and forms an enzyme-substrate intermediate^46^. GPI is then transferred to the newly exposed C-terminus of the protein^3^ (Fig. 1a, Step 13). The deletion of every subunit caused the inactivation of GPI-TA, and GPI-APs were not synthesized (Fig. 2).

#### GPI-anchor remodeling

PGAP1 is a GPI-inositol deacylase that removes an acyl chain from the inositol ring of GPI^47^ (Fig. 1a, Step 14). Subsequently, a side chain EtNP attached to the second Man is removed by PGAP5^48^ (Fig. 1a, Step 15). Although KO of *PGAP1* or *PGAP5* causes delayed transport of GPI-APs from the ER to the Golgi^47,48^, it did not affect the expression of GPI-AP on the cell surface at a steady state (Fig. 2).

In the Golgi apparatus, GPI fatty acid remodeling occurs, in which an unsaturated fatty acid at the sn-2 position of the GPI lipid is replaced with a saturated fatty acid. Elimination of an unsaturated fatty acid and transfer of a saturated fatty acid are mediated by PGAP3^49,50^ (Fig. 1a, Step 17) and PGAP2^51^ (Fig. 1a, Step 18), respectively. The surface expression of GPI-APs in *PGAP3*-KO cells was only mildly affected, whereas in *PGAP2*-deficient cells, the surface expression of GPI-APs, except prion, was significantly reduced (Fig. 2). This is because, in *PGAP2*-deficient cells, lyso-GPI-APs having only one hydrocarbon chain are transported to the cell surface, but they are unstable and released from the plasma membrane. The expression of prion in *PGAP2*-KO cells was almost comparable with that in WT cells, which was analyzed below.

Glycan moieties in some GPI-APs are further modified in the Golgi. PGAP4 transfers GalNAc to Man1 via 1,4-linkage^12,13^ (Fig. 1a, Step 19). B3GALT4 could transfer Gal to the GalNAc residue^14^ (Fig. 1a, Step 20). The KO cells did not affect the expression of GPI-APs (Fig. 2).

#### Shedding of GPI-APs from the cell membrane

GPI-APs can be cleaved at GPI moieties and released from the membrane as intact proteins (Fig. 1a). Angiotensin-converting enzyme (ACE), glycerol phosphodiesterase 2 (GDE2) (also known as GDPD5), GPI-specific phospholipase D (GPI-PLD/GPLD1), and PGAP6 have GPI-cleaving enzyme (GPIase) activity on specific GPI-APs^24^. We also constructed gene knockout cells to study the biological function of specific GPI-APs. The surface expression levels of CD59 in *PGAP6*-KO cells, CD55 in *GPLD1*-KO, and CD55 and prion in *GDPD5*-KO and *ACE*-KO cells were mildly but significantly higher than that in WT cells (Fig. 2). In the WT cells, a fraction of those GPI-APs would be released constantly by those GPIases. By the gene-KO, the surface expression was increased because the GPI-APs are not released.

### KO of genes encoding enzymes involved in GPI biosynthesis led to partial PI-PLC resistance

**Fig. 3:**
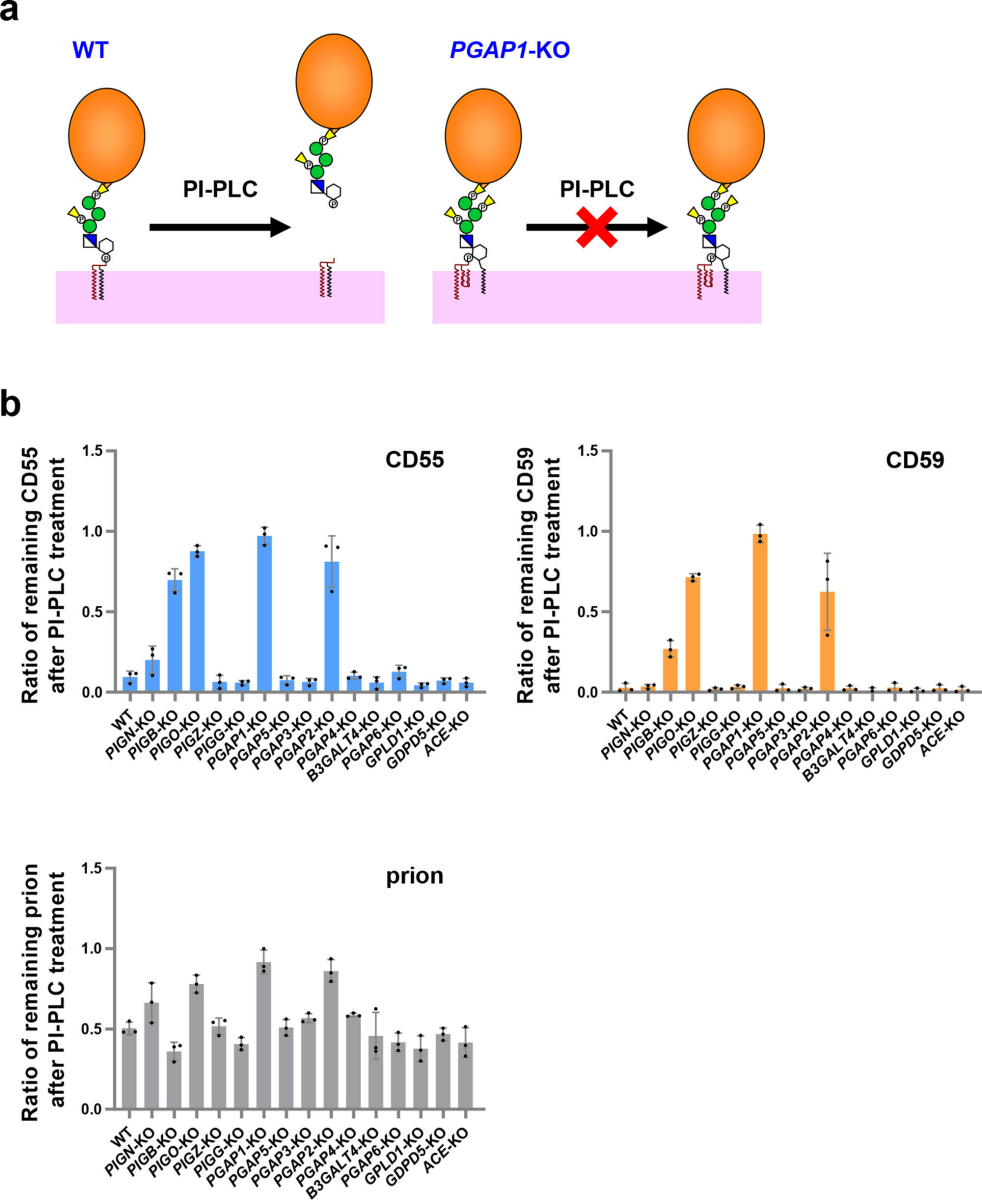
The PI-PLC catalytic reaction releases GPI-APs on the cell surface. **a** Schematic representation of PI-PLC activity against GPI-APs. The remodeled mature GPI-APs on the cell surface in WT cells are cleaved by PI-PLC and released from the plasma membrane. On the other hand, the inositol-acylated GPI-APs in *PGAP1*-KO cells show resistance to PI-PLC. **b** PI-PLC sensitivity of CD55, CD59, and prion. WT and KO cell lines were treated with and without PI-PLC. The residual amounts of CD55, CD59, and prion on the cell surface after PL-PLC treatment were analyzed by flow cytometry. The ratio of remaining GPI-APs was calculated and is shown as the mean ± SD from three independent experiments.

PI-PLC hydrolyzes PI at the site between the phosphate and glycerol backbone^10,52^. Via treatment with PI-PLC, GPI-APs on the cell surface are cleaved and released (Fig. 3a). When an acyl chain is modified to the inositol ring, the GPI-APs show resistance to PI-PLC.

Among the GPI-KO cell library, KO cells that still show GPI-APs on the cell surface were picked up and used to detect the PI-PLC sensitivity of GPI-APs with different GPI structures. In the WT cells, more than 90% of CD55 and CD59 were cleaved by PI-PLC, whereas approximately 50% of the prion proteins showed resistance to PI-PLC treatment (Fig. 3b), suggesting that half of the prion expressed on the cell surface of HEK293 cells has an additional acyl chain. By KO of *PGAP1*, which encodes GPI-inositol deacylase, GPI-APs showed complete resistance to PI-PLC (Fig. 3b). In the *PGAP2*-KO cells, the majority of GPI-APs were not cleaved by PI-PLC (Fig. 3b), suggesting that GPI-APs retained on the cell surface in *PGAP2*-KO cells are inositol-acylated. Since it is known that inositol-acylated GPI-APs cannot be a substrate for PGAP3^50^, GPI-APs that are not processed by PGAP1 may be expressed as inositol-acylated GPI-APs having three hydrocarbon chains in *PGAP2*-KO cells. In other GPI remodeling-deficient cells, including *PGAP5*-KO, *PGAP3*-KO, *PGAP4*-KO, and *B3GALT4*-KO cells, GPI-APs are processed by PI-PLC at a similar efficiency in WT cells. Similarly, cells defective in GPI-cleaving enzymes (including *PGAP6*-KO, *GPLD1*-KO, *GDPD5*-KO, and *ACE*-KO cells) showed that GPI-APs were as sensitive to PI-PLC as WT cells.

To determine the correlation of GPI-inositol deacylation with the presence of side chain EtNPs and Mans on GPI structures, we used cells defective in *PIGN*, *PIGB*, *PIGO*, *PIGZ*, or *PIGG* for GPI-AP cleavage by PI-PLC. In *PIGZ*-KO and *PIGG*-KO cells, the PI-PLC sensitivity of GPI-APs was normal, suggesting that the fourth Man and the second side chain EtNP on GPI do not affect GPI-inositol deacylation. On the other hand, in *PIGO*-KO cells, GPI-APs on the cell surface were resistant to PI-PLC treatment. KO of *PIGB* or *PIGN* mildly reduced PI-PLC sensitivity. These results suggest that the GPI structure affects GPI-inositol deacylation.

### Dissection of inositol-acylated GPI on prion

**Fig. 4:**
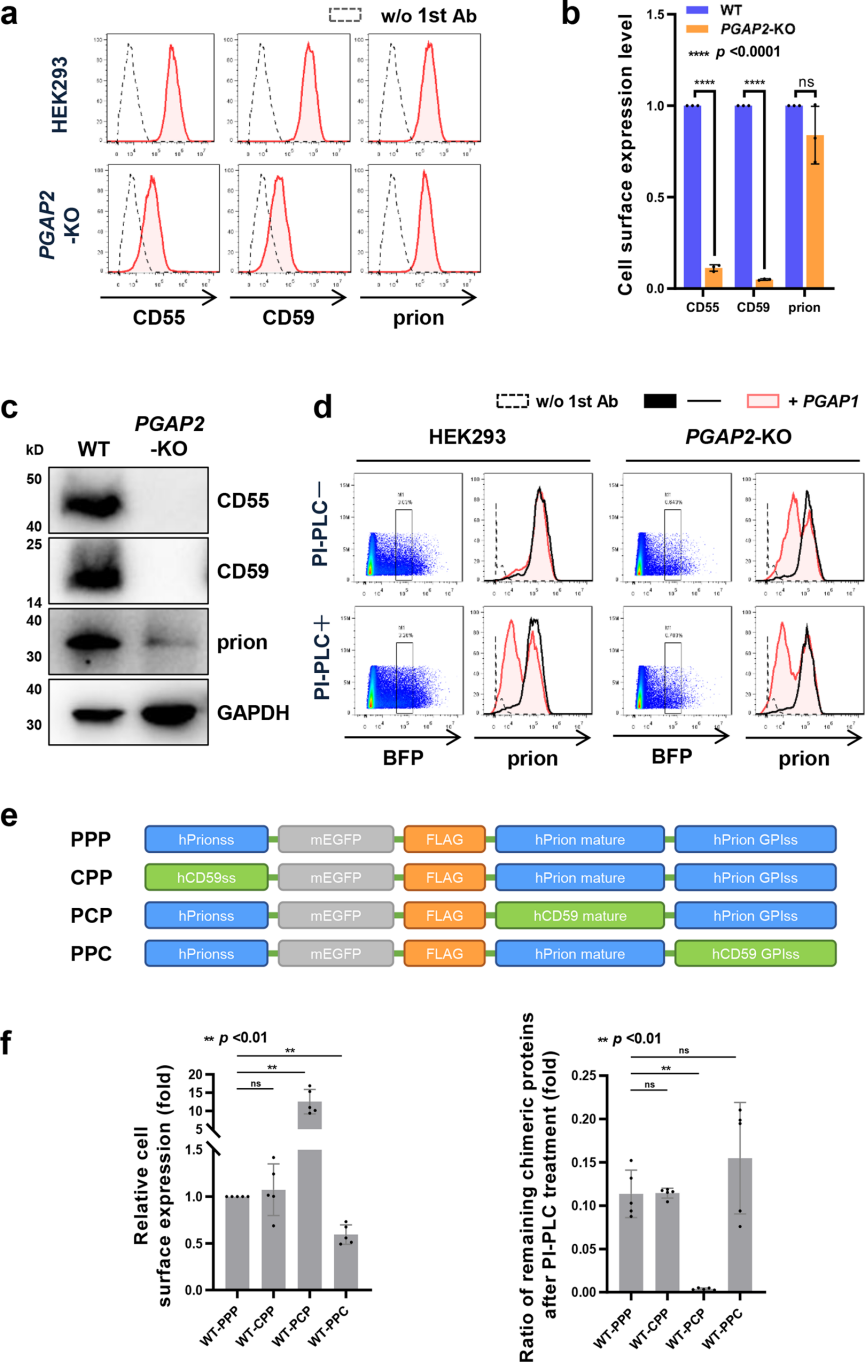
Fractions of prion proteins in HEK293 cells possess inositol-acylated GPI. **a** Expression of CD55, CD59, and prion on the surface in WT and *PGAP2*-KO cells was analyzed by flow cytometry. Red shaded areas indicate the expression of GPI-APs in WT and *PGAP2*-KO cells and dashed lines show the background (stained without 1st antibody). **b** The mean fluorescence intensities of CD55, CD59, and prion in WT cells were set as 1, and the relative intensities of those in *PGAP2*-KO cells are displayed as the mean ± SD from three independent experiments with *p* values (Student’s *t* test). ns not significant. **c** Cell lysates prepared from WT and PGAP2-KO cells were analyzed by western blotting. CD55, CD59, and prion were detected. GAPDH was used as a loading control. **d** Flow cytometric analysis of prion proteins in WT and *PGAP2*-KO cells overexpressing *PGAP1*. A plasmid expressing *PGAP1* or empty vector was transiently transfected into WT and *PGAP2*-KO cells together with a pME-BFP plasmid. Three days after transfection, cells were harvested and treated with or without PI-PLC. The BFP-expressing cells were gated, and the surface expression of prion was analyzed. Red shaded areas indicate cells expressing *PGAP1*, black solid lines indicate cells expressing empty vector, and dashed lines show background (stained without 1st antibody). **e** Schematic representation of EGFP-FLAG-tagged prion proteins. In the PPP construct, all the parts corresponding to the N-terminal signal, mature part, and GPI attachment signal consist of prion parts. In the CPP, PPC, and PCP constructs, the N-terminal signal, GPI attachment signal, and mature part of the PPP were replaced with CD59 signals. **f** Cell surface expression of EGFP-FLAG-tagged chimeric proteins was analyzed by flow cytometry. The plasmids pLIB2-PPP-IRES2-mBFP, pLIB2-PPC-IRES2-mBFP, pLIB2-PCP-IRES2-mBFP, or pLIB2-CPP-IRES2-mBFP were stably transfected into HEK293 cells. Cells were harvested and treated with or without PI-PLC. The BFP-expressing cells were gated, and the surface expression of PPP, PPC, PCP, or CPP was analyzed. The mean fluorescence intensities of PPP in HEK293 cells were set as 1, and the relative intensities (mean ± SD values) of other chimeric proteins from five independent experiments were plotted (left panel). HEK293 cells expressing chimeric proteins were treated with and without PI-PLC. The residual amounts of chimeric proteins on the cell surface after PI-PLC treatment were analyzed by flow cytometry. The ratios of remaining proteins (mean ± SD values from five independent experiments) were plotted (right panel). **p < 0.001 according to an unpaired Student’s *t* test. ns not significant.

In the initial analysis of GPI-AP expression and PI-PLC sensitivity in the GPI-KO cell library, the prion protein behaved differently from other GPI-APs, such as CD55 and CD59 (Figs. 2 and 3b). In *PGAP2*-KO cells, CD55 and CD59 surface expression decreased by more than 90%, whereas prion proteins on the cell surface were only 20% reduced on average (Fig. 4a and b). The *PGAP2*-rescued cells restored the reduction in CD59 and CD55 (Supplementary Fig. 5). According to western blotting of cell lysates, CD59 and CD55 were not detectable in *PGAP2*-KO cells, whereas prion proteins could be detected (Fig. 4c). Approximately, 50% of prion proteins were cleaved by PI-PLC in WT cells, whereas in *PGAP2*-KO cells, 85% of prion proteins on the cell surface showed resistance against PI-PLC (Fig. 3b), suggesting that prion proteins have inositol-acylated GPI anchors.

To determine whether PI-PLC resistance of prion proteins is caused by inefficient inositol deacylation in HEK293 cells, we transiently overexpressed *PGAP1* encoding a GPI-inositol deacylase in WT and *PGAP2*-KO cells to facilitate inositol deacylation (Fig. 4d). The sensitivity of prion proteins to PI-PLC increased when PGAP1 was overexpressed, and at the same time, prion expression was significantly reduced in *PGAP2*-KO cells. This confirms that most of the prion protein on the surface of HEK293 cells is present without inositol deacylation. Compared with CD55 and CD59, PGAP1 seems to have a lower affinity with prion protein, which leads to the presence of prion protein without inositol deacylation in *PGAP2*-deficient cells.

To determine regions of the prion protein that cause inefficient deacylation by PGAP1, we constructed EGFP-FLAG-tagged prion chimera proteins, in which the N-terminal ER insertion signal sequence, mature protein, or C-terminal GPI attachment signal was replaced with corresponding regions of CD59 (Fig. 4e). Compared to endogenous prion proteins, the EGFP-FLAG-tagged chimeric constructs showed high PI-PLC sensitivity (Fig. 4f). Nevertheless, PPP (prion signal sequence/prion mature/prion GPI-attachment signal), CPP (CD59 signal sequence/prion mature/prion GPI-attachment signal), and PPC (prion signal sequence/prion mature/CD59 GPI-attachment signal) constructs showed around 11–15% resistance to PI-PLC in WT cells. On the other hand, the PCP (prion signal sequence/CD59 mature/prion GPI attachment signal) construct on the cell surface was almost completely cleaved by PI-PLC. These results indicate that the mature part of the prion protein determines unique PI-PLC resistance, probably its structure is not efficiently recognized by the GPI-inositol deacylase PGAP1. Alternatively, it is possible that a lower % of PI-PLC sensitivity is caused by the prion dimer formation since it has been reported that prion proteins form dimer structures^53^.

### GPI-KO library for determination of GPI signatures recognized by aerolysin

**Fig. 5:**
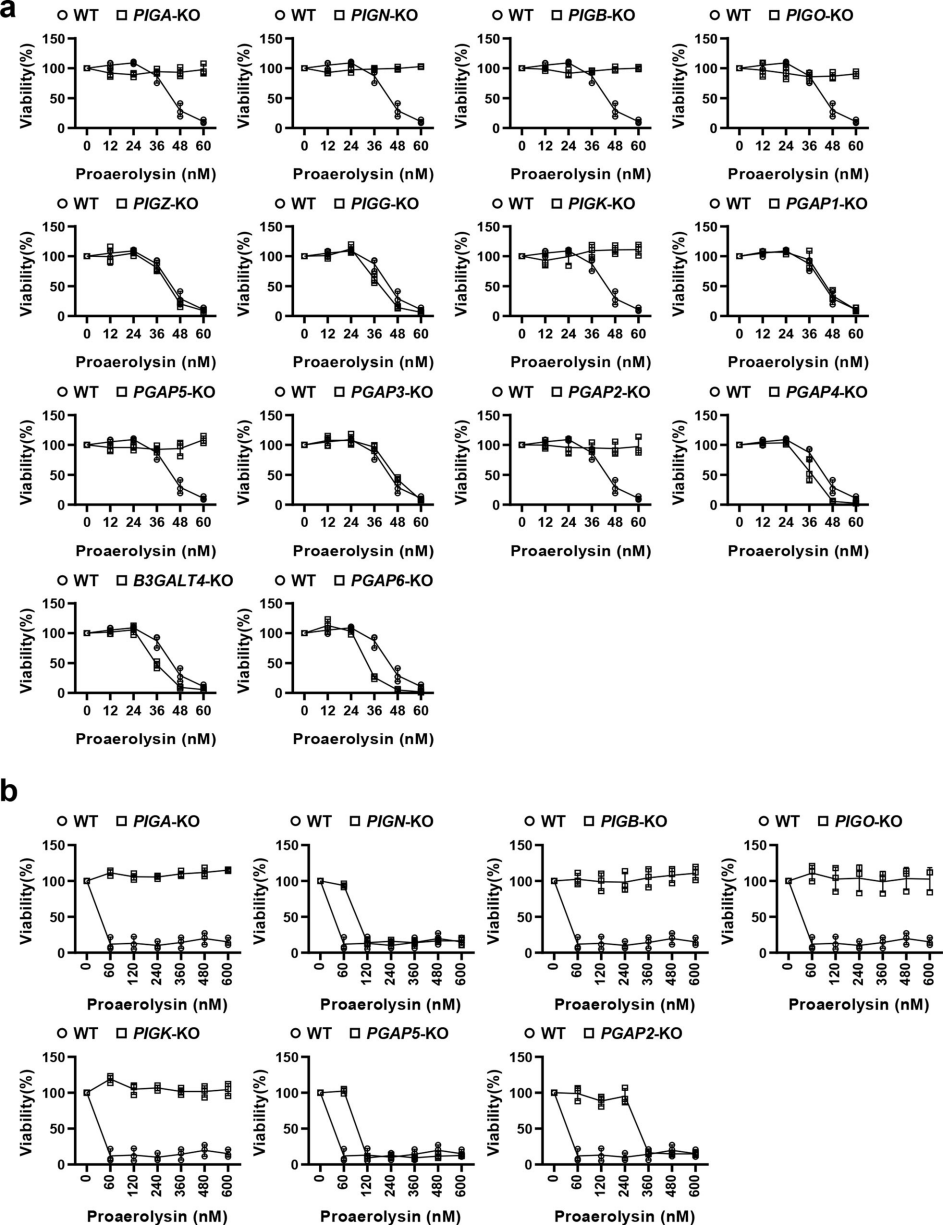
Aerolysin recognizes glycan-remodeled GPI structures by PGAP5. **a** Fourteen GPI-KO cell lines were treated with different concentrations of proaerolysin (0, 12, 24, 36, 48, and 60 nM), and cell viability was analyzed by Cell Counting Kit-8 (CCK-8, MedChemExpress). HEK293 WT cells were used as a control. The cell viability of cells treated without proaerolysin was set as 1, and the relative viability was plotted as the mean ± SD from three parallel experiments. **b** Cell lines that showed resistance to aerolysin at low concentrations (60 nM) were selected and treated with high concentrations of proaerolysin (up to 600 nM). The cell viability was plotted as described above.

Aerolysin^54^ is a member of the pore-forming toxin family secreted from *Aeromonas hydrophila*, which causes gastroenteritis, deep wound infection, and septicemia. Aerolysin binds to GPI-APs to target cells, and the C-terminal peptide of aerolysin is cleaved off by cell surface proteases, forming an activated heptameric complex, which is inserted into the membrane and acts as a channel^55^. Therefore, cell lines defective in GPI biosynthesis showed resistance to aerolysin^56^. Although it has been reported that the GPI-glycan structure and N-glycan structures on GPI-APs are required for toxin binding^57,58^, the precise GPI signature recognized by aerolysin remains unclear. To determine the GPI structures that aerolysin recognizes, we used a GPI-KO cell library to compare the sensitivity of 15 cell lines to aerolysin (Fig. 5a, b). WT HEK293 cells are sensitive to aerolysin, and almost all cells died at ≥60 nM. On the other hand, the cell lines with weak or no expression of GPI-APs on the cell surface, such as *PIGA*-, *PIGB*-, *PIGO*-, and *PIGK*-deficient cells, showed resistance to aerolysin treatment (Fig. 5b). In *PIGK*-deficient cells, although non-protein-linked free GPIs are expressed on the cell surface, aerolysin does not bind with them, showing hyper resistance to aerolysin^57,59^. The *PIGN*- and *PGAP2*-deficient cells also showed resistance to aerolysin, even if GPI-APs were expressed at low levels (Fig. 2). On the other hand, *PGAP5*-deficient cells express GPI-APs on the cell surface at the same level as WT cells. However, the cells showed resistance to aerolysin (Fig. 5a). *PGAP5*-KO cells showed resistance to aerolysin at 60 nM and then showed sensitivity at higher concentrations (Fig. 5b). The phenotype was rescued by the expression of *PGAP5* in *PGAP5*-KO cells (Supplementary Fig. 6). The results indicate that the presence of EtNP on Man2 negatively affects the binding of aerolysin to GPI-APs. In addition, *PGAP6*-deficient cells are highly sensitive to aerolysin, probably due to the increased expression of GPI-APs on the cell surface. The slightly high sensitivity of *PGAP4*- and *B3GALT4*-deficient cells to aerolysin means that the glycan side chain modifications of GPI weaken aerolysin binding. These observations indicate that the GPI-KO library is useful to clarify the signature of GPI for toxin recognition.

## Discussion

Due to the complex biosynthetic pathway and variable side chain modifications of GPI anchors, it is difficult to compare how gene knockout impacts GPI-AP expression and function. Here, we established and utilized a knockout cell library to analyze the surface expression of GPI-APs and their sensitivity to PI-PLC and to determine the structural signature of GPIs recognized by aerolysin. In addition, we showed that prion proteins have unique properties, the majority of which was resistance to PI-PLC in HEK293 cells. In this study, we constructed a knockout cell library consisting of single-gene knockout cell lines. Since we have already validated a set of gRNAs against each GPI biosynthetic gene, the current knockout cell library could be established using these materials. It is possible to generate complicated GPI structures via a combination of the library with the gRNAs.

When the effects of gene-KO on GPI biosynthesis in mammalian cells are compared with those in yeast cells, similar and different phenomena are observed. It is known that yeast *GPI1*, which is the mammalian *PIGQ* homolog, is a non-essential gene among the GPI-GnT genes, suggesting that GPI-GnT is still active in *gpi1*-deficient yeast cells^60^. The *PIGQ*-KO still showed the expression of GPI-APs, consistent with yeast *GPI1*. On the other hand, KO of mammalian *PIGZ*, which encodes GPI-ManT-IV, did not affect the surface expression of GPI-APs, whereas the yeast homolog *SMP3* is essential for the GPI biosynthetic pathway. The difference would be due to the different substrate recognition of the mammalian and yeast GPI-EtNP transferase-III (mammalian PIGO and PIGF or yeast Gpi13 and Gpi11)^61^. The GPI-KO cell library could be utilized for the complementation analysis of homologous genes in different species.

The knockout cell library of GPI biosynthetic genes enables us to produce GPIs with unique structures and functions. In particular, when KO cells defective in genes required for GPI glycan and lipid remodeling are used, we can evaluate the functions of GPI side chains and lipid moieties. In *PGAP5*-KO cells, a side chain EtNP remains attached to the second Man on GPI. Although the expression of GPI-APs was compatible with that in WT cells, the KO cells showed resistance to aerolysin, providing evidence that aerolysin recognizes the second Man without modification. Furthermore, the GPI-KO cell library would be useful to assay the effect of GPI structures on the conversion of prion proteins from cellular forms to scrapie forms. Some reports suggest the importance of GPI-anchors on prion structural changes and pathogenicity^62–64^. It would be possible to check the efficiency of prion structural changes dependent upon GPI structures by culturing KO cells with the scrapie forms.

When a GPI attachment signal is added to the C-terminus of secretory proteins or extracellular regions of type-I membrane proteins, the proteins can be expressed as GPI-APs. Therefore, the GPI-anchoring system is useful to tether the target proteins on the cell surface (Supplementary Fig. 7a). In addition, it is known that many GPI-APs are incorporated into extracellular vesicles, such as exosomes and virus-like particles (VLPs) (Supplementary Fig. 7b). It has been reported that various immunomodulatory proteins, antigens, and single-chain variable fragments are expressed as GPI-APs and loaded on VLPs to enhance immune responses, vaccination, and targeting, respectively^65–67^. Another unique feature is that purified GPI-APs can be inserted into the cell membrane through their lipid moieties in vitro. This is called GPI cell painting^68^ (Supplementary Fig. 7c), which enables the addition of the characteristics into the targeted cells. When recombinant proteins are expressed as GPI forms, the KO cell library produces recombinant proteins with different GPI structures, which might affect protein behaviors, including incorporation into exosomes or VLPs and cell painting. The KO cell library provides opportunities to analyze various phenomena related to GPI-APs.

In short, the knockout cell library of GPI biosynthetic genes can become a sustainable resource for exploring various applications and methods of GPI-AP biology and can provide insights into the genetic and biosynthetic regulation of GPI.

## Methods

### Cell lines, antibodies, and reagents

HEK293 (ATCC CRL-1573) cells and their derivative cells were cultured in Dulbecco’s modified Eagle’s medium (DMEM) containing 10% fetal bovine serum (FBS) at 37 °C with 5% CO_2_. *PGAP1*-KO^69^ and *PGAP2*-KO^70^ cells have been constructed previously. KO cell lines used in this study are listed in Supplementary Table 1. Mouse monoclonal anti-CD55 (clone IA10)^50^, anti-CD59 (clone 5H8)^50^, anti-CD230 (14-9230-82; Thermo Fisher Scientific), anti-CD109 (556039; BD Biosciences), anti-GAPDH (60004-1-Ig, clone 1E6D9; Proteintech), anti-FLAG (F3165; M2; Sigma-Aldrich) and rabbit monoclonal anti-HA (3724S; Cell Signaling Technology) were used as primary antibodies. F(ab’)2-goat anti-mouse IgG, PE (12-4010-82; Thermo Fisher Scientific) and F(ab’)2-donkey anti-rabbit IgG, PE (12-4739-81; Thermo Fisher Scientific), and Goat Anti-Mouse IgG, HRP (HS201-1; TransGen Biotech) were used as the secondary antibodies. For flow cytometric analysis, antibodies were used at 10 µg/ml. For western blotting, the primary antibodies and the secondary antibodies were used at 1 and 0.2 µg/ml, respectively. PI-PLC (Thermo Fisher Scientific) and proaerolysin (produced and purified in the laboratory) were used for treatments. Cell Counting Kit-8 (CCK-8, MedChemExpress) was used to detect cell viability.

### Plasmids

The gRNA sequences were designed on the E-CRISP website^71^ and ligated into the pX330-EGFP vector^72^. All gRNA sequences used for gene KO in this study are listed in Supplementary Table 2. pME-Hyg-3FLAG-rPGAP1^69^ and pME-Hyg-3HA-hPGAP5^48^ were used for rescue experiments. All the primers used in this study are listed in Supplementary Table 3. The DNA fragments corresponding to *PGAP2*, *PGAP6*, *PGAP4*, and *B3GALT4* were amplified from cDNA prepared from HEK293 cells using the primers in Supplementary Table 3. pME-Hyg-3HA-hPGAP2 was generated by in-fusion cloning of full-length *PGAP2* cDNA into the SalI/NotI site of pME-Hyg-HA. pME-Hyg-hPGAP6-3HA was obtained by in-fusion cloning of full-length *PGAP6* cDNA into the XhoI/MluI site of pME-Hyg-HA. pLIB2-BSD-hPGAP4 was obtained by in-fusion cloning of full-length *PGAP4* cDNA into the EcoRI/NotI site of pLIB2-BSD. pLIB2-BSD-hB3GALT4 was obtained by in-fusion cloning of full-length *B3GALT4* cDNA into the EcoRI/NotI site of pLIB2-BSD. The DNA fragments corresponding to the N-terminal ER-insertion signal, mature protein or C-terminal GPI-attachment signal of prion or CD59 and EGFP-FLAG tag were amplified from prion cDNA and pME-Neo2dH-ssCD59-EGFP-FLAG-CD59. The DNA fragments consisting of PPP (ssprion-EGFP-FLAG-prion-prionss), PPC (ssprion-EGFP-FLAG-prion-CD59ss), PCP (ssprion-EGFP-FLAG-CD59-prionss), or CPP (ssCD59-EGFP-FLAG-prion-prionss) were ligated into the EcoRI/NotI site of the vector pLIB2-IRES2-mBFP to generate pLIB2-PPP-IRES2-mBFP, pLIB2-PPC-IRES2-mBFP, pLIB2-PCP-IRES2-mBFP and pLIB2-CPP-IRES2-mBFP. The DNA fragments corresponding to a mature part and a C-terminal GPI attachment signal of TEX101, GPC3, CRIPTO and SPACA4 were amplified from human cDNA clones by PCR, and they were spliced with ssCD59-HA from pME-puro-ssCD59-HA-CD14. Then, the DNA fragments corresponding to HA-tagged GPI-APs were ligated into the EcoRI/NotI of pLIB2-IRES2-mBFP, generating pLIB2-ssCD59-HA-TEX101-IRES2-mBFP, pLIB2-ssCD59-HA-GPC3-IRES2-mBFP, pLIB2-ssCD59-HA-CRIPTO-IRES2-mBFP and pLIB2-ssCD59-HA-SPACA4-IRES2-mBFP.

### Establishment of KO cell lines

The pX330-EGFP plasmids containing gRNA sequences were transfected into HEK293 cells. Three days after transfection, the cells with a high EGFP fluorescence signal were sorted with a cell sorter S3 (Bio-Rad). The collected cells were cultured for more than one week, diluted, and transferred to a 96-well plate for culture to obtain monoclonal knockout cells. Gene knockout was analyzed by Sanger sequencing, and clonal cells without the WT allele were selected.

### PI-PLC treatment and flow cytometric analysis

Cells (~10^6^ cells/well) were harvested and washed with 500 μl of PBS. The samples were mixed with reaction buffer (5 U/ml PI-PLC, 0.5% bovine serum albumin (BSA), 5 mM EDTA, and 10 mM HEPES in DMEM without fetal calf serum) and incubated at 37 °C for 1.5 h. After washing the incubated cells with PBS, the cells were stained with primary antibodies (10 µg/ml) (anti-CD55, anti-CD59, or anti-CD230) in FACS buffer (PBS containing 1% BSA and 0.1% NaN3) for 25 min on ice. The samples were then washed twice with FACS buffer and stained with the secondary antibody (10 µg/ml) (F(ab’)2-goat anti-mouse IgG) in FACS buffer for 25 min on ice. After incubation, the samples were washed twice with FACS buffer and analyzed using Accuri C6 (BD). The data were analyzed using Accuri C6 and FlowJo software (BD).

### Transfection and retrovirus-based infection

Cells (~10^6^ cells/well) were plated in 6-well plates 1 day before transfection. For transient transfection, 4 μg of plasmids were transfected into cells using polyethyleneimine MAX (PEI-MAX) (Polysciences). Three days after transfection, the transfected cells were analyzed. For retrovirus-based infection, HEK293 cells (~10^6^ cells) were transfected with 1 μg of pGP, 1 μg of pLC-VSVG, and 2 μg of pLIB2-IRES2-mBFP containing the target gene using PEI-MAX. After 12-16 h, the culture medium was changed, and the cells were cultured for 24 h. Then, the culture medium was collected, filtered with a 0.22 μm filter, and mixed with the same amount of DMEM containing 16 μg/ml hexadimethrine bromide (Sigma). The medium containing retrovirus was incubated with receiver cells overnight. One week after infection, cells were used for analysis.

### Proaerolysin treatment and cell viability assay

Cells (2 × 10^4^ cells/well) were cultured in a 96-well plate for one day. After removing the medium, 200 μl of prewarmed (at 37 °C) medium containing proaerolysin at different concentrations was added to the 96-well plate. After 8 h of incubation at 37 °C, cell viability was measured using a CCK-8 kit. Each sample was measured in triplicate. Cell viability (%) was calculated as (proaerolysin-treated cells (*A*_450_) − background (*A*_450_))/(nontreated cells (*A*_450_) − background (*A*_450_)) × 100.

### Cell lysate preparation and analysis

Cells (~10^6^ cells/well) were lysed with 100 μl of RIPA buffer and protein inhibitor cocktail (EDTA-Free, MedChemExpress) on ice for 30 min. After incubation, the samples were centrifuged at 10,000×*g* for 10 min at 4 °C to remove insoluble fractions. The supernatants were mixed with sample buffer and kept at 4 °C overnight. The protein samples were subjected to sodium dodecyl sulfate-polyacrylamide gel electrophoresis and western blotting. Original western blotting images are shown in Supplementary Fig. 8.

### Statistics and reproducibility

Statistical analyses were done using GraphPad Prism8 (GraphPad Prism Software) and Microsoft Excel 2016 (Microsoft). Source data are deposited as Supplementary data 1. For the statistical analyses, at least three independent or parallel experiments were performed. Unpaired Student’s *t* test was used to evaluate comparisons between two individual groups. *P* value < 0.05 were considered statistically significant.

### Reporting summary

Further information on research design is available in the Nature Research Reporting Summary linked to this article.

## Supplementary information

Supplementary Information

Description of Supplementary Files

Supplementary Data 1

Reporting Summary

## Data Availability

The authors declare that data supporting the findings of this study are available within the paper and its supplementary information files.
