# Supplementary material for: A knockout cell library of GPI biosynthetic genes for functional studies of GPI-anchored proteins
Source: Commun Biol. 2021 Jun 23;4:777. doi: 10.1038/s42003-021-02337-1 (PMC8222316; doi:10.1038/s42003-021-02337-1)
Supplement: Supplementary file 1 — Supplementary Information [file 42003_2021_2337_MOESM1_ESM.pdf]

Supplementary Fig. 1

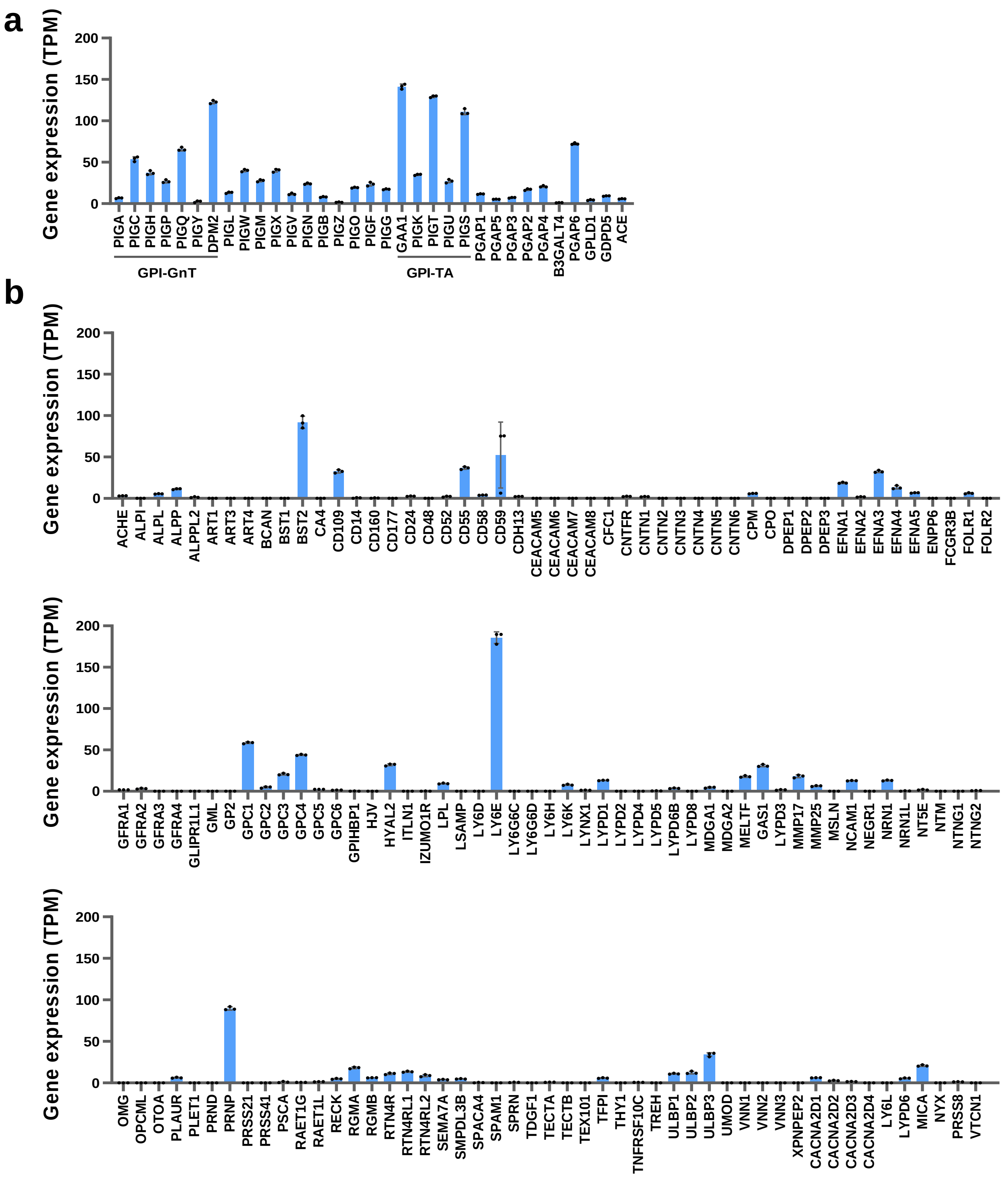

Supplementary Fig. 1 Expression of GPI biosynthetic genes and GPI-AP genes in HEK293 cells.

a Expression of GPI biosynthetic genes in HEK293 cells was analyzed using RNA-seq data in Huang et al., *Dev. Cell* (2021) 8: 1195-1209. All the genes required for GPI biogenesis were expressed in HEK293 cells, while the expression of a few genes, such as PIGY, PIGZ and B3GALT4, was restricted. **b** Expression of genes encoding GPI-APs in HEK293 cells. At least 67 GPI-APs were predicted to be expressed in HEK293 cells (TPM value ≥ 1). TPM, transcript per million. The gene expression (mean ± SD from three independent experiments) were displayed in bar plots.

Supplementary Fig. 2

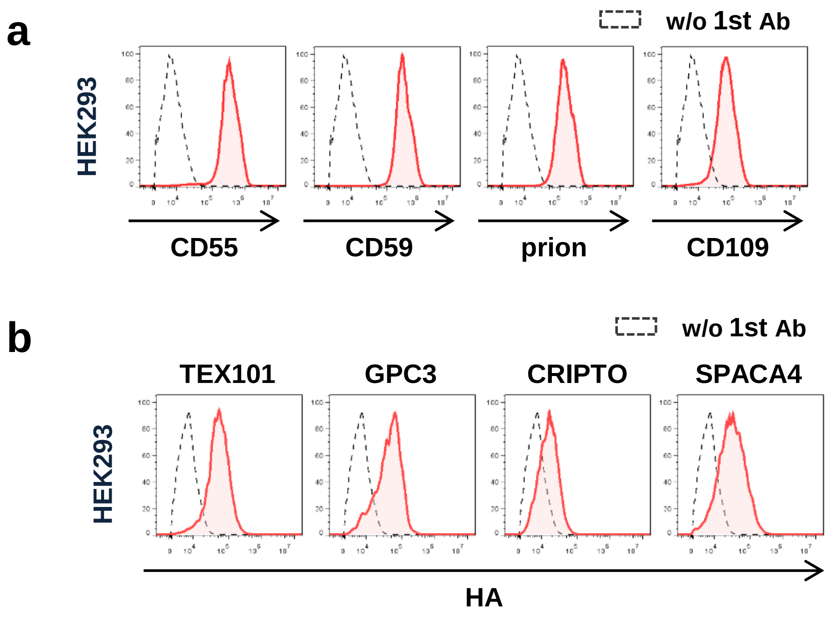

Supplementary Fig. 2 Evaluation of the ability to synthesize various GPI-APs in HEK293 cells.

a Surface expression of endogenous CD55, CD59, prion and CD109 was analyzed by flow cytometry.

b The vectors pLIB2-ssCD59-HA-TEX101-IRES2-mBFP, pLIB2-ssCD59-HA-GPC3-IRES2-mBFP, pLIB2-ssCD59-HA-CRIPTO-IRES2-mBFP or pLIB2-ssCD59-HA-SPACA4-IRES2-mBFP were stably infected into WT cells. The BFP-expressing cells were gated and analyzed for exogenous expression of GPI-APs by flow cytometry.

Supplementary Fig. 3

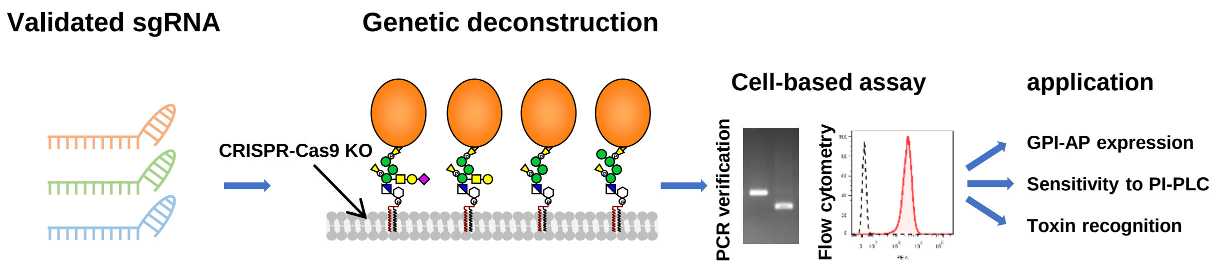

Supplementary Fig. 3 Construction of the knockout cell library of GPI biosynthetic genes. A set of single guide RNAs (sgRNAs) against genes involved in GPI biosynthesis, remodeling and cleavage was designed and validated. Using CRISPR-Cas9 technology, the target genes were knocked out in HEK293 cells to construct a knockout cell library showing different GPI-AP expression levels and structures. This library can be used to analyze the impact of GPI-AP expression and sensitivity to PI-PLC and to determine aerolysin recognition.

Supplementary Fig. 4

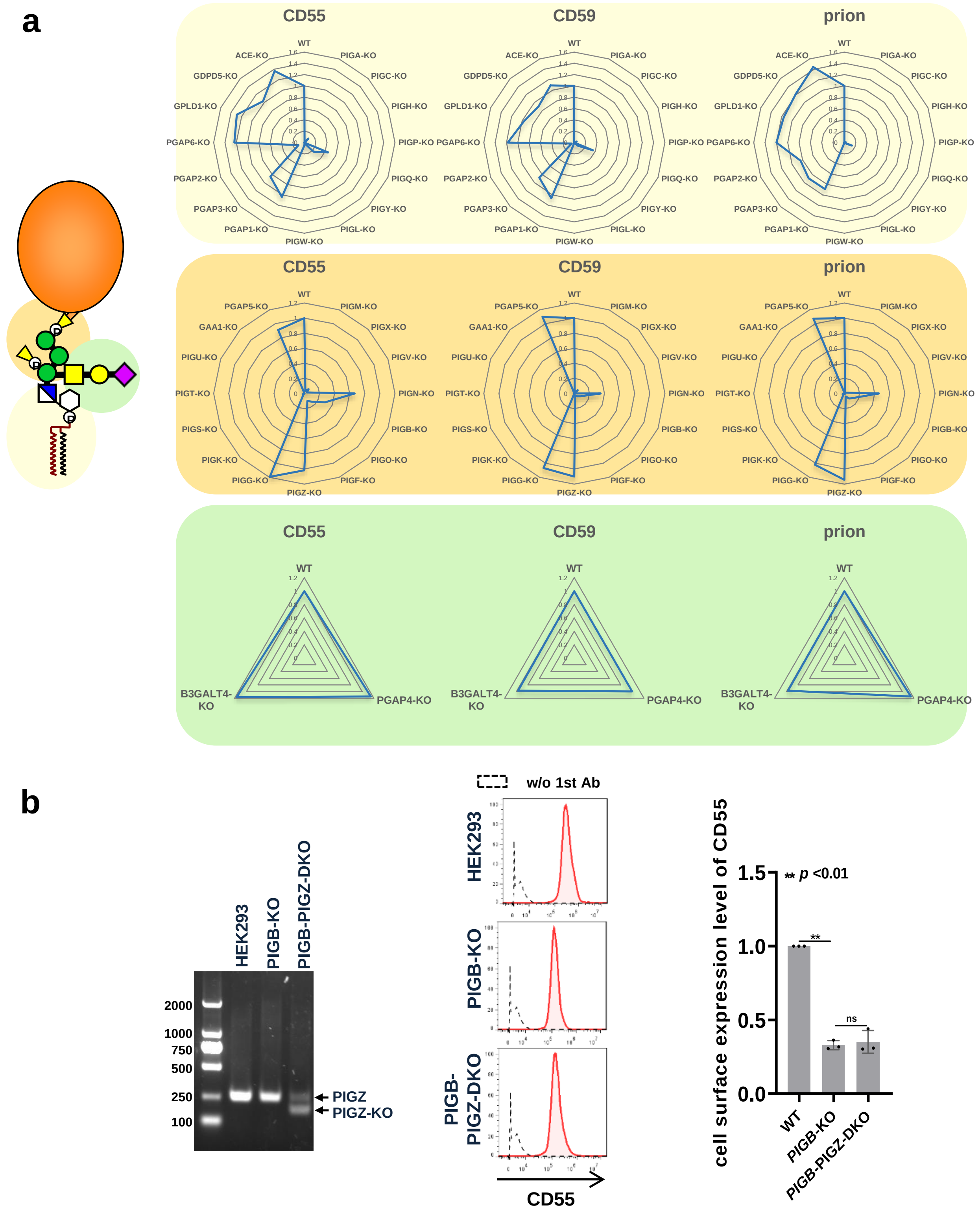

Supplementary Fig. 4 Cell surface expression of GPI-APs in GPI-KO cell library.

a Cell surface expression of three endogenous GPI-APs, CD55, CD59, and prion, shown in Fig. 2 was used. The GPI-KO cell library was categorized into three groups a: modification and remodeling of PI (including initial GPI biosynthetic steps, lipid remodeling, and GPI cleavage) shown in yellow, GPI core glycan synthesis and attachment to proteins shown in orange, and GPI side-chain glycan synthesis shown in green. The expression level of GPI-APs in WT cells was set as 1, and the relative mean ± SD values from three independent experiments were plotted in radar charts.

b *PIGZ* was further knocked out in *PIGB*-KO cells, and the knockout efficiency was verified by PCR (left). Cell surface expression of CD55 was detected by flow cytometry (middle). The expression level of CD55 in WT cells was set as 1, and the relative mean ± SD values from three independent experiments were displayed in a bar plot (right). **, $p < 0.01$ according to an unpaired Student's t-test.

Supplementary Fig. 5

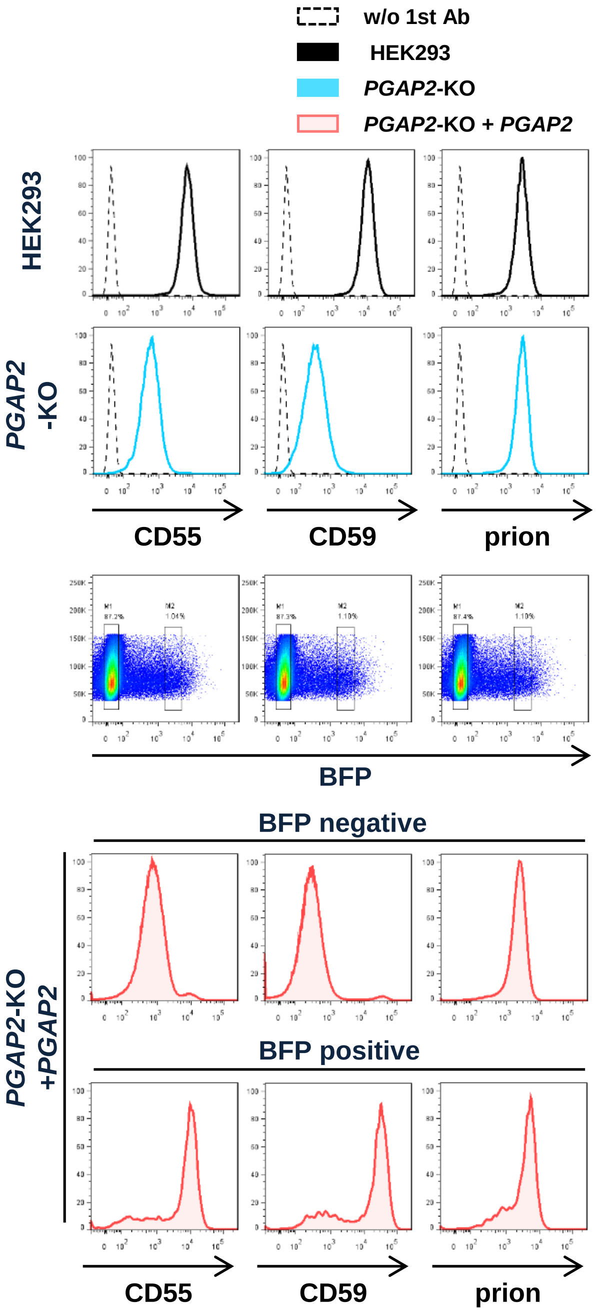

Supplementary Fig. 5 Rescue of the phenotype in *PGAP2*-KO cells.
PGAP2-KO cells were transiently transfected with both a plasmid expressing *PGAP2* and a plasmid expressing BFP. The BFP-negative and BFP-positive cells were gated, and the surface expression of CD55, CD59, and prion was analyzed in each gate. The surface expression of these GPI-APs in WT and *PGAP2*-KO cells was analyzed as a control.

Supplementary Fig. 6

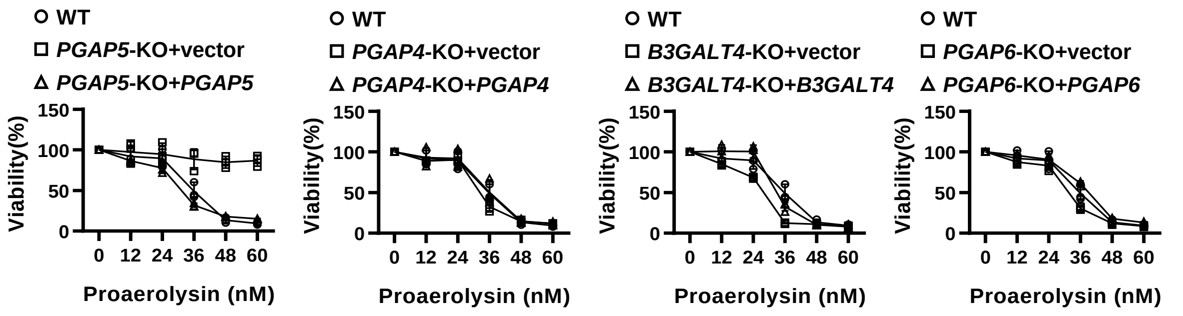

Supplementary Fig. 6 Restoration of aerolysin sensitivity in *PGAP5*-KO, *PGAP4*-KO, *B3GALT4*-KO and *PGAP6*-KO cells.

PGAP5-KO, *PGAP4*-KO, *B3GALT4*-KO and *PGAP6*-KO cells were restored by stable expression of the responsible genes. Aerolysin sensitivity was assayed using the restored cells.

Supplementary Fig. 7

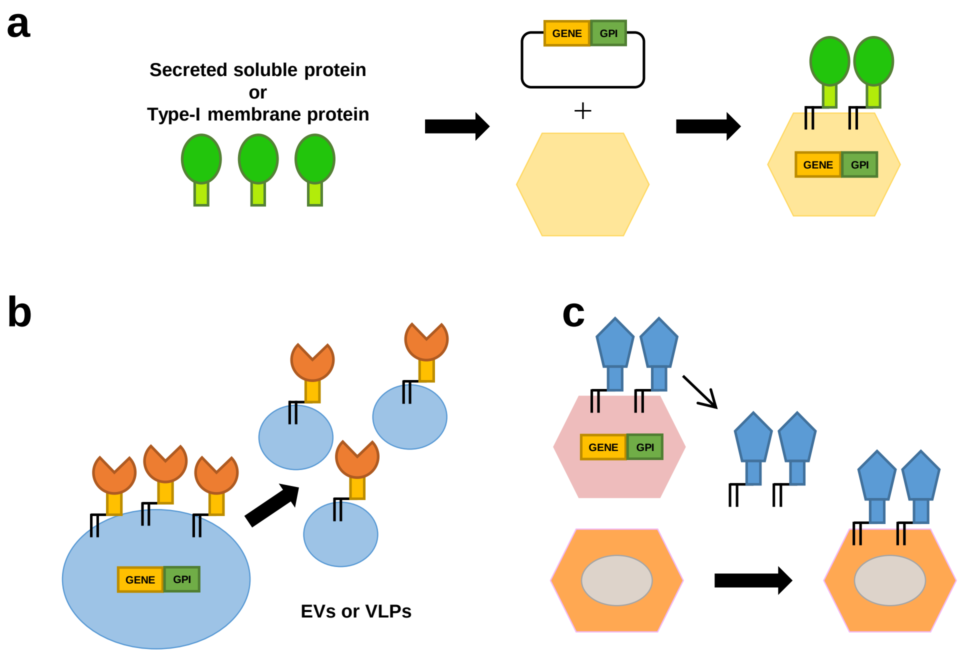

Supplementary Fig. 7 Applications of the GPI-KO cell library.
Since the GPI-KO library can synthesize GPIs with various structures, it can be applied to research using GPI-APs.
a Expression of secreted soluble proteins and type-I transmembrane proteins as recombinant GPI-APs. Soluble or type-I proteins can be expressed as GPI-anchored forms. For secreted soluble proteins, a GPI attachment signal is added to the C-terminus. For type-I membrane proteins, the C-terminal transmembrane region is replaced with a GPI attachment signal.
b Production of exosomes (EVs) or virus-like particles (VLPs) with specific GPI structures. It has been reported that GPI-APs are incorporated into EVs and VLPs. Functional proteins of interest can be expressed as GPI-anchored forms and then expressed in EVs or VLPs.
c GPI-AP cell painting. It has been reported that purified GPI-APs are spontaneously inserted into the cell membrane, and they can decorate the cell surface and change cell properties.

Supplementary Fig. 8

a Fig. 4c

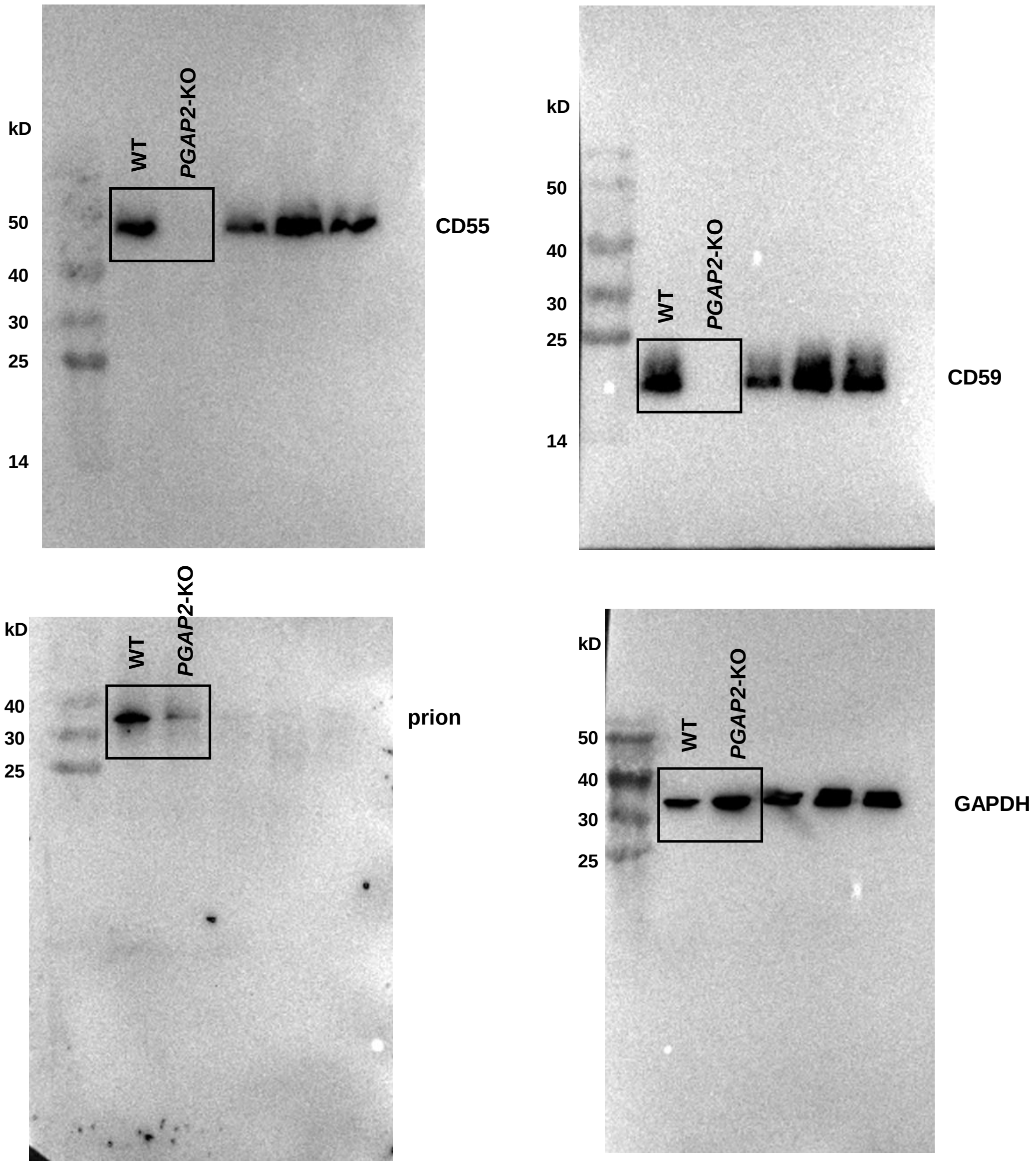

b Fig. S4b

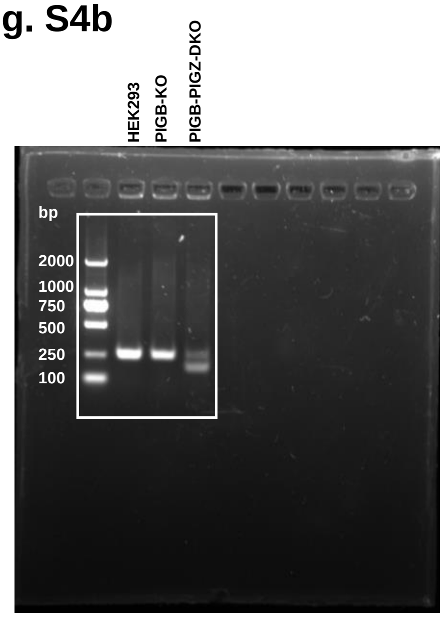

Supplementary Fig. 8 Source data.
a Original uncropped western blotting data of Fig. 4c.
b An uncropped gel image of Fig. S4b.

Supplementary Table 1

Cell Lines	
HEK293	ATCC CRL-1573
ACE-KO	This study
B3GALT4-KO	This study
GAA1-KO	This study
GDPD5-KO	This study
GPLD1-KO	This study
PGAP1-KO	(Liu et al., 2018)
PGAP2-KO	(Matabaro et al., 2017)
PGAP3-KO	This study
PGAP4-KO	This study
PGAP5-KO	This study
PGAP6-KO	This study
PIGA-KO	This study
PIGB-KO	This study
PIGC-KO	This study
PIGF-KO	This study
PIGG-KO	This study
PIGH-KO	This study
PIGK-KO	This study
PIGL-KO	This study
PIGM-KO	This study
PIGN-KO	This study
PIGO-KO	This study
PIGP-KO	This study
PIGQ-KO	This study
PIGS-KO	This study
PIGT-KO	This study
PIGU-KO	This study
PIGV-KO	This study
PIGW-KO	This study
PIGX-KO	This study
PIGY-KO	This study
PIGZ-KO	This study

Supplementary Table 2

	Target1 sequence	Target2 sequence	Check primer1	Check primer2	Indel	Clone No.
ACE	GCACGACACCAACATCACCG NGG	GCTGCCGCCGCAGCCCGCCC NGG	AGAAGGGGCAGAGCCGAG CA	AGCAGCCACCCTCATCCAT CCAAC	-147	6
B3GALT 4	GTGGGGGTAAGTGCCCGACG NGG	GCCACTGCTCCTCTGATACG NGG	CTGGTATCAGAGCTGGTCTT GCGAGGG	CCGTCAGAGCCACCCACCA GCTT	-158<br>-157	6
GAA1	GACCCAGCGCACTTACATGT NGG	GCGGTGGGCGGCGAAGTCCC NGG	GCACTGAGGGTATCAGGCC CA	TGCGTGTAGACCTCCAGCC CTACT	-78<br>-86	1
GDPD5	GCTCGTAGTACTGCAGGGGC NGG	GGCAGCCGTAGATGCCCGTG NGG	ACAAGCTGGGGGCCTCCAT GG	TGACCCTGTTCCAGGGTGG CTCA	-42	8
GPLD1	GCCTGGCCTGCTGATCATGT NGG	GGATTGATACAGTTTCCCCT NGG	GTGACCTGCTTAGAGAGAA GCGGTG	CCTCTCGTTCACTGAGCCTC TCACT	-161<br>-159	3
PGAP3	GCGGTTGGTCCTGCTAGCTG NGG	GACTCATGTAGATTGGCTGG NGG	GTGGAAGCTTCATACTCCTA AGCTCCTC	ACCCTGGTCTGATTTCTGTG CCAT	-125	1
PGAP4	GAAGTCGGTGACAGGCCAGG NGG	GATGATGGTGATCACCAGCC NGG	ATGAGCACTTCAACCTCTCC AGCTG	CGATTGGCCACAGGGACAT ACTTGG	-182<br>-181	2
PGAP5	GTCAACAGCGTGGCGCTGAA NGG	GGCTGAGTGAGCAACCGAAG NGG	CCCTTCGATCGTGTCTTAGA CAGAACTC	GCAGAGGCCCAGGTCCACA	-135	2
PGAP6	GGCCGGGCTGGCACCGGGAC NGG	GCTCTTCCCGCTGTAGCCGG NGG	AGAGGCGGAGGCGGCAGA GGC	ACAGGTGCGCCCGGGGTGT CACAAT	-83<br>-79	4
PIGA	GAGAGAGTGTAGCTGAGGCA NGG	GGCATCCGTTACCTCACCAG NGG	CAGGTAATAGAGGACACAT CTCTTAACTGGGTT	CATTGTCTTGGCGTGGAAG AGAGCA	+12<br>-212<br>-234	2
PIGB	GCCCCTAAGCAAGTGCGGAA NGG	GCTTTATCTTGCCGTGGGAG NGG	ACTTAATGGATGGACGAGC AGCGC	TGTTGAAGTACAAGGTAGAC TTTCTCTTTCGCA	+ 576	3
PIGC	GGAGCTCTTCCAGGAATCGC NGG	GCTGGATACAATGGCAGCAT NGG	ATGTATGCTCAACCTGTGAC TAACACCAA	GAAGGCATGCAGGGACCGG G	+35<br>-413	1
PIGF	GATCTCTCCATGTACCCTGC NGG	GGTCATAGCTTAGGTCTCTT NGG	CTGCCCAGCAGTCTTTCAGT TGGTA	CACAACGGTCAAGGCATGT AATGAAAACA	+276<br>-253	3
PIGG	GCAACAGGTAGCGAAAGTCC NGG	GCTCTGGCAGAGGAACGAAC NGG	ACGTGACGCCACTGTCGCT G	AAGTGGGGCCAGTAGAGGT GGTTG	-77<br>-78	4
PIGH	GCTACTACTCCCCGTCCTGC NGG	GAGGGGGTTCGGCTTTGGGA NGG	TCATGGAGGATGAGCGGAG CTTTTC	GGAAGGGGAATGTACTATC CAGGAGCA	-156	4
PIGK	GACACTTCTATAAACAGAAA NGG	GTCGATAATTAAACCAGAAT NGG	TGCAGCTCCATGGTTAGCC TTGAT	AGGGTTATTTTCACTGAAGT TCAAGGTTAAGC	-35	8
PIGL	GCGTTGGCGGTCTTGGCATG NGG	GTACACCCAGTGCCTTAGGC NGG	CTACTGCGCAGGCTCAGTG CT	GTTTCTCTGCTCCTCAATCC TGCTGG	-155	5
PIGM	GCCACACCAAAGACGCCGGC NGG	GTGAGGAGCCAACCCAGCAG NGG	ATGGGCTCCACCAAGCACT GG	GCGACGAGTCTTTTCTTTAT CAAGTAGAGGA	-174	6
PIGN	GGAGTCATTCCATGAACCAA NGG	GTATTGTTGAAGCTGATAAC NGG	CACTGGATATGCTGCTGTTC TTTACTTTGG	GGGAAATTAGGACAATTTAG CTTAAGCAGCT	-370<br>-334	4
PIGO	GGGCAATGCCAGCGTAGAAG NGG	GCAAGGAGCTTAGTTTGCCC NGG	ATGCAGAAAGCCTCAGTGTT GC	AGCTGCTTAATGAGATTGTC TTCCACT	-256	1
PIGP	GCTCTTTCTGGCAATGGCGA NGG	GTGACAAGGGAAGAATACAT NGG	ATGGTGCCACGGAGCACAT CG	TGTCTCTCCACCGTGCTCC CTG	-124	2
PIGQ	GCCGGAGCAGAGCAGCGCCG NGG	GTGACAGCAACACCTGGCGC NGG	CCCACGTGCTGCGTCTCGA	ACCTCACTGCGTGCTACCG TG	-313	3
PIGS	GAGCGGTAGCCCCAGCACGA NGG	GGGCATTCAGGCCACTGATC NGG	TCAGACGAGATTCCCTTCAG GCTTG	CACATTTCAGAGGAATCTCT CTTTCATGC	-67	4
PIGT	GTCATCACCCCGCTGCCTTC NGG	GATTCGGAGCTTCAGCGGGA NGG	AGCGGAAGTCACTTCTCAC ACGC	CTTGGAGATCAGCTGCCCC AG	-62<br>-61	3
PIGU	GGTGGTGGCTGTGACAGTGC NGG	GGACACCACCTCCACCCGCT NGG	TTTCTGGGAGCCGTAGTCC CCA	CCCCAGGGCCCAAACCTCA	-48<br>-53	15
PIGV	GCCCCAGGACCCATCCCGGA NGG	GCACATTCAGGACAAAGGAT NGG	AGGCCTGAGGGAGCTCAAT CCT	ACTTCTTACTGCCTAGTCCA GTCATGTAGC	-70	2
PIGW	GCATTCTGTATCCTGTGCAG NGG	GTGTAATTATCTTTGGGGCA NGG	ATGTCTGAAAAGCAGATGAA GGAAGCT	ATTGCTCCTGTCCCATAGAG CTCA	-165	2
PIGX	GCTCACGCGCGGGCCCGCCG NGG	GCGCGGGACCTAGATCAGTA NGG	ATAAATCTGGGGCAGCGCG C	ACGCTTGACGGCTGACTTT CCT	-137	4
PIGY	GGAATAAGAACAGTCAACGT NGG	GGATGGGTATTAAACTCTTC NGG	GGGAATAGCTTATCCAATCA TTGATGGGATCCC	AGTCCTAAGCCTGATATGC GCAAAGCAAA	-180	13
PIGZ	GACACACTGGTTAACAAAGG NGG	GGATTTCTTAGCCTTAGGGG NGG	ATGGCGGTGCAGTCTACTC CAGG	CATTCTCCACTGTCATTGTC TGAGTCCAGG	-78	1

Supplementary Table 3

Primer name	Sequence (5′–3′)
PGAP2-F	GTGCCTGATTACGCTGGCGCCGTCGACTACCAGGTCCCACTACCACTGGATCGGG
PGAP2-R	TTCTCTAGACTAGTCTAGCGGCCGCTCAGAATCGCTTTTCCTCAGGCTGAGAGGT
PGAP4-F	CTCCTTCTCTAGGCGCCGGAATTCCCACCATGAGCACTTCAACCTCTCCAGCT
PGAP4-R	TCTCGAGCCTAGGGGCCGAGGCGGCCGCTTAGAGGAGACTGGGATGAAAG
B3GALT4-F	CTCCTTCTCTAGGCGCCGGAATTCCCACCATGCAGCTCAGGCTC
B3GALT4-R	TCGAGCCTAGGGGCCGAGGCGGCCGCTTAGCTCTGAAGCCAGG
PGAP6-F	CTGCGGAATTCCGGAATTTCCTCGAGCCACCATGGGCCGGGCTGGCACCG
PGAP6-R	CGTCATAGGGGTATCCGCCACGCGTCGTCACTGCGTACAGTTCCTCCCGA
pME-F	CCCTGCTTGCTCAACTCTACGTCTT
pME-R	TAATTCGAGCTCGGTACCCG
ssCD59/prion-F	CCTCACTCCTTCTCTAGGCGCCGG
ssCD59/prion-R	CCAGGCTTCGGGCGCTTCTTAAGCTTGTCATCGTCGTCCTTGTAGTCCTGC
prion-F	AAGAAGCGCCCGAAGCCTGGAGG
prion-R	CACTAGGGGCCGAGGCGGCCGCTCATCCCACTATCAGGAAGATGAGGAAAGAGATCAGG
M-prion-R	TCCCACCATTTCTCTGGTAATAGGCCTGAGATTCC
CD59ss-F	CCAGAGAAATGGTGGGACATCCTTATCAGAGAAA
CD59ss-R	ACTAGGGGCCGAGGCGGCCGCTTAGGGATGAAGGCTCCAGGCTGCTGCCA
PILB2-F	CCTCCATCCGCCCCGTCTCTCC
PILB2-R	CCTCACATTGCCAAAAGACGGCAATATGGTGG
CD59-HA-F	CACTCCTTCTCTAGGCGCCGGAATTCCCACCATGGGAATCCAAGGA
CD59-HA-R	CTCGAGGCCGGCAAGCTTTCCA
CRIPTO-F	TTGCCGGCCTCGAGCTGGGCCATCAGGAATTTGCTCG
CRIPTO-R	GGGGCCGAGGCGGCCGCTTAATAGTAGCTTTGTATAGAAAGGCAGATGCCAAC
SPACA4-F	CTTGCCGGCCTCGAGGTCAAGGACTGCGTCTTCTGTGAGC
SPACA4-R	AGGGGCCGAGGCGGCCGCTTATCACAGCAAACGTGGAGGAAGCAG
TEX101-F	AGCTTGCCGGCCTCGAGCTGTATTGTCAAAAGGGTCTGTCCATGACTG
TEX101-R	CTAGGGGCCGAGGCGGCCGCTTAGGAAAAGTGAATAAATG
GPC3-F	CTTGCCGGCCTCGAGGGACAGGCGCAGCCCCCGCCGCCGC
GPC3-R	TAGGGGCCGAGGCGGCCGCTTACTAGTGCACCAGGAAGAAGAAGCAC
