# Supplementary material for: A knockout cell library of GPI biosynthetic genes for functional studies of GPI-anchored proteins
Source: Commun Biol. 2021 Jun 23;4:777. doi: 10.1038/s42003-021-02337-1 (PMC8222316; doi:10.1038/s42003-021-02337-1)
Supplement: Supplementary file 2 — Description of Supplementary Files [file 42003_2021_2337_MOESM2_ESM.pdf]

Description of Additional Supplementary Files

File name: Supplementary Data 1

Description: Source data for plots used in Figures.
