# Supplementary material for: A knockout cell library of GPI biosynthetic genes for functional studies of GPI-anchored proteins
Source: Commun Biol. 2021 Jun 23;4:777. doi: 10.1038/s42003-021-02337-1 (PMC8222316; doi:10.1038/s42003-021-02337-1)
Supplement: Supplementary file 4 — Reporting Summary [file 42003_2021_2337_MOESM4_ESM.pdf]

nature research

Corresponding author(s): Morihisa Fujita

Last updated by author(s): Apr 28, 2021

Reporting Summary

Nature Research wishes to improve the reproducibility of the work that we publish. This form provides structure for consistency and transparency in reporting. For further information on Nature Research policies, see our Editorial Policies and the Editorial Policy Checklist.

Statistics

For all statistical analyses, confirm that the following items are present in the figure legend, table legend, main text, or Methods section.

n/a	Confirmed	
☐	☒	The exact sample size (*n*) for each experimental group/condition, given as a discrete number and unit of measurement
☐	☒	A statement on whether measurements were taken from distinct samples or whether the same sample was measured repeatedly
☐	☒	The statistical test(s) used AND whether they are one- or two-sided<br>*Only common tests should be described solely by name; describe more complex techniques in the Methods section.*
☒	☐	A description of all covariates tested
☐	☒	A description of any assumptions or corrections, such as tests of normality and adjustment for multiple comparisons
☐	☒	A full description of the statistical parameters including central tendency (e.g. means) or other basic estimates (e.g. regression coefficient) AND variation (e.g. standard deviation) or associated estimates of uncertainty (e.g. confidence intervals)
☐	☒	For null hypothesis testing, the test statistic (e.g. *F*, *t*, *r*) with confidence intervals, effect sizes, degrees of freedom and *P* value noted<br>*Give P values as exact values whenever suitable.*
☒	☐	For Bayesian analysis, information on the choice of priors and Markov chain Monte Carlo settings
☒	☐	For hierarchical and complex designs, identification of the appropriate level for tests and full reporting of outcomes
☒	☐	Estimates of effect sizes (e.g. Cohen's *d*, Pearson's *r*), indicating how they were calculated

Our web collection on statistics for biologists contains articles on many of the points above.

Software and code

Policy information about availability of computer code

Data collection	Flow cytometric data were collected using BD Accuri C6 controlled by Accuri C6 software.<br>Western blotting images were obtained by Tanon 5200 Multi.
Data analysis	Flow cytometric data were analyzed by BD FlowJo 7.6.<br>Statistical analysis were performed using GraphPad Prism 8 and Microsoft Excel 2016.

For manuscripts utilizing custom algorithms or software that are central to the research but not yet described in published literature, software must be made available to editors and reviewers. We strongly encourage code deposition in a community repository (e.g. GitHub). See the Nature Research guidelines for submitting code & software for further information.

Data

Policy information about availability of data

All manuscripts must include a data availability statement. This statement should provide the following information, where applicable:

- Accession codes, unique identifiers, or web links for publicly available datasets
- A list of figures that have associated raw data
- A description of any restrictions on data availability

All the data and relevant materials including primers, reagents and cells, that support the findings of this study are available from the corresponding author upon reasonable request.

Field-specific reporting

Please select the one below that is the best fit for your research. If you are not sure, read the appropriate sections before making your selection.

☒ Life sciences ☐ Behavioural & social sciences ☐ Ecological, evolutionary & environmental sciences

For a reference copy of the document with all sections, see nature.com/documents/nr-reporting-summary-flat.pdf

Life sciences study design

All studies must disclose on these points even when the disclosure is negative.

Sample size	Flow cytometric data were from three independent replicates. Western blotting was performed in two independent experiments. Cell viability assay was from at least two independent experiments, and each experiment was performed in three parallel experiments. The representative results are shown.
Data exclusions	No data were excluded.
Replication	All of flow cytometric analyses were performed at least three times. Western blotting and cell viability assay were performed at least two times. Experimental results are reliably reproduced.
Randomization	Not relevant to this study because the paper does not include comparison studies among group.
Blinding	The investigators were not blinded during data collection.

Reporting for specific materials, systems and methods

We require information from authors about some types of materials, experimental systems and methods used in many studies. Here, indicate whether each material, system or method listed is relevant to your study. If you are not sure if a list item applies to your research, read the appropriate section before selecting a response.

Materials & experimental systems

n/a	Involved in the study	
☐	☒	Antibodies
☐	☒	Eukaryotic cell lines
☒	☐	Palaeontology and archaeology
☒	☐	Animals and other organisms
☒	☐	Human research participants
☒	☐	Clinical data
☒	☐	Dual use research of concern

Methods

n/a	Involved in the study	
☒	☐	ChIP-seq
☐	☒	Flow cytometry
☒	☐	MRI-based neuroimaging

Antibodies

Antibodies used	The following antibodies were used in this study:<br>Mouse monoclonal anti-CD55 (clone IA10), anti-CD59 (clone 5H8), anti-CD230 (14-9230-82; Thermo Fisher Scientific), anti-CD109 (556039; BD Biosciences), anti-GAPDH (60004-1-Ig, clone 1E6D9; Proteintech), anti-FLAG (F3165; M2; Sigma-Aldrich), rabbit monoclonal anti-HA (3724S; Cell Signaling Technology), F(ab')2-goat anti-mouse IgG, PE (12-4010-82; Thermo Fisher Scientific) and F(ab')2-donkey anti-rabbit IgG, PE (12-4739-81; Thermo Fisher Scientific), Goat Anti-Mouse IgG, HRP (HS201-1; TransGen Biotech).
Validation	All antibodies used in this study have been validated.<br>anti-CD55, anti-CD59, anti-CD230 and anti-CD109 antibodies were validated by the gene knockout cells and cells knocked out GPI pathway genes. anti-FLAG and anti-HA antibodies have been validated by tagged protein overexpression.<br>anti-GAPDH, F(ab')2-goat anti-mouse IgG, PE, F(ab')2-donkey anti-rabbit IgG, PE, and Goat Anti-Mouse IgG, HRP were validated by multiple publications. Detailed information could be found on the manufactures' website.<br>anti-GAPDH, http://www.ptgcn.com/products/GAPDH-Antibody-60004-1-Ig.htm<br>F(ab')2-goat anti-mouse IgG, https://www.thermofisher.com/cn/zh/antibody/product/Goat-anti-Mouse-IgG-H-L-Secondary-Antibody-Polyclonal/12-4010-82<br>F(ab')2-donkey anti-rabbit IgG, https://www.thermofisher.com/cn/zh/antibody/product/Donkey-anti-Rabbit-IgG-H-L-Secondary-Antibody-Polyclonal/12-4739-81<br>Goat Anti-Mouse IgG, HRP, https://www.transgen.com.cn/antibody_second/403.html

Eukaryotic cell lines

Policy information about cell lines

Cell line source(s)	human embryonic kidney 293 (HEK293) cell line (ATCC CRL-1573)
Authentication	None of the cell lines were authenticated
Mycoplasma contamination	Original parental HEK293 cell line was confirmed as negative for mycoplasma contamination. The knockout cell lines derived from the HEK293 cells were not tested for mycoplasma contamination.
Commonly misidentified lines (See ICLAC register)	None of the cell lines used are listed in the ICLAC database.

Flow Cytometry

Plots

Confirm that:

☒ The axis labels state the marker and fluorochrome used (e.g. CD4-FITC).

☒ The axis scales are clearly visible. Include numbers along axes only for bottom left plot of group (a 'group' is an analysis of identical markers).

☒ All plots are contour plots with outliers or pseudocolor plots.

☒ A numerical value for number of cells or percentage (with statistics) is provided.

Methodology

Sample preparation	Cells (~10^6 cells/well) were harvested and washed with 500 µl of PBS. After washing the incubated cells with PBS, the cells were stained with primary antibodies (10 µg/ml) in FACS buffer (PBS containing 1% BSA and 0.1% NaN3) for 25 min on ice. The samples were then washed twice with FACS buffer and stained with the secondary antibody (10 µg/ml) in FACS buffer for 25 min on ice. After incubation, the samples were washed twice with FACS buffer, and were analyzed within 1 hour. For PI-PLC treatment, the samples were mixed with reaction buffer (5 U/ml PI-PLC, 0.5% BSA, 5 mM EDTA, and 10 mM HEPES in DMEM without FCS) and incubated at 37°C for 1.5 hours before cell staining.
Instrument	BD Accuri C6
Software	BD Accuri C6 software was used to collect data; and FlowJo was used to analyze the data.
Cell population abundance	We only used cultured cell lines, so cell population abundane was always 100%. At least 20000 cells were analyzed for each sample.
Gating strategy	We used cell lines and gating of different populations does not apply to this study.

☐ Tick this box to confirm that a figure exemplifying the gating strategy is provided in the Supplementary Information.
